# A bench study on the prevention of lung-to-lung aspiration with double-lumen endobronchial tubes and bronchial blockers

**DOI:** 10.1038/s41598-023-50792-z

**Published:** 2024-01-02

**Authors:** Hyerim Kim, Taikyung Seol, Sung-Hee Han, Honghyeon Kim, Jin-Young Hwang

**Affiliations:** 1 Department of Anesthesiology and Pain Medicine, SMG-SNU Boramae Medical Center, College of Medicine, Seoul National University, Boramaero 5-Gil, Dongjakgu, Seoul, 07061 Republic of Korea; 2 Department of Anesthesiology and Pain Medicine, Sheikh Khalifa Specialty Hospital, RAK, United Arab Emirates; 3 Department of Anesthesiology and Pain Medicine, Seoul National University Bundang Hospital, College of Medicine, Seoul National University, Seoul, Republic of Korea; 4 Department of Anesthesiology and Pain Medicine, Seoul National University Hospital, Seoul, Republic of Korea

**Keywords:** Anatomy, Medical research

## Abstract

Effective lung isolation prevents lung-to-lung aspiration during thoracotomy for the management of hemoptysis. Double-lumen endobronchial tubes (DLT) and bronchial blockers are commonly used for lung separation during thoracic surgery. In this study, the fluid-sealing characteristics of the endobronchial cuffs of three different commercially available DLTs (Broncho-Cath with a polyvinylchloride cuff, Broncho-Cath with a polyurethane cuff, and Human Broncho with a silicone cuff) and two different bronchial blockers (Arndt and Coopdech bronchial blockers) were evaluated using a benchtop model. The lateral decubitus position for the surgical management of bleeding from the right lung was simulated. The artificial tracheobronchial tree was placed horizontally, with the left bronchus in the dependent position and the right bronchus in the non-dependent position. In the DLT experiments, the tracheobronchial tree was intubated with left-sided DLTs, and the endobronchial cuff was inflated to maintain the intracuff pressure at 25 cmH_2_O. In the experiments with bronchial blockers, each bronchial blocker was inserted into the right bronchus, and the endobronchial cuff was inflated to seal the main bronchus. A fluid leakage test around the endobronchial cuff was performed using three different types of DLT (size 35, 37, and 41 Fr, each) and two different types of bronchial blockers (9 Fr). The 5 mL of colored water was poured into the right bronchus to simulate the blood flow from the operative side, and the times to the first and 100% leakage around the endobronchial cuff were recorded. Each bronchial blocker showed significantly less leakage over time than the other DLTs (*P* < 0.05). Fluid was not fully leaked around the cuffs for 24 h with either bronchial blocker. The times to first and 100% leakage were not significantly different among different types of DLTs. The times to first and 100% leakage did not also differ among the three different sizes of each type DLT. There was no significant difference in the time to first leakage around the endobronchial cuffs between Arndt and Coopdech bronchial blockers. Bronchial blockers provided a more effective seal against lung-to-lung aspiration than DLTs in the lateral decubitus position for thoracotomy in the benchtop model.

## Introduction

Massive hemoptysis is a potentially life-threatening condition that can result from various causes, such as pulmonary diseases, coagulopathy, or trauma^1^. Protection of the airway is the initial step in the management of hemoptysis. Effective isolation of the bleeding segment prevents aspiration of blood into the large airways and contralateral lung and maintains airway patency^2^. An emergent thoracotomy for surgical intervention is sometimes required to manage hemoptysis^3^. Thoracic surgery is commonly performed in the lateral decubitus position, where the non-dependent operative side collapses to facilitate surgical exposure and the dependent lung is ventilated. During thoracotomy for managing hemoptysis, the bleeding side is placed over the normal lung in the lateral decubitus position. Therefore, if blood drains into the airways of the normal lung from the surgical bleeding site, oxygenation and ventilation can be interrupted.

Double-lumen endobronchial tubes (DLT) and bronchial blockers are commonly used for lung separation during thoracic surgery^4^. Inflation of the endobronchial cuff of the DLT or bronchial blocker allows separation of the ventilation and helps prevent spillage of contaminated material and blood into the ventilated lung. The endobronchial cuff of a DLT is a high-volume, low-pressure cuff with a diameter larger than that of the main bronchus. When the cuff is inflated within the bronchus, longitudinal and transverse folds occur and these folds form channels for shedding blood into the ventilated lung^5,6^. With DLTs, the endobronchial cuff is generally inflated with 1–2 mL of air, and its intracuff pressure should be maintained at 20–30 cmH_2_O to avoid mucosal ischemic injury. Compared with DLTs, a larger volume of air (4–8 mL) is required to inflate the endobronchial cuff and seal the main bronchus, thereby resulting in a higher intracuff pressure^7^.

The endobronchial cuffs of DLTs and bronchial blockers have traditionally been made of polyvinylchloride (PVC) plastic. Recently, DLTs with endobronchial cuffs made of polyurethane or silicone have been introduced. Whether the cuff material affects the sealing characteristics of the endobronchial cuff has not been determined. Furthermore, the fluid-sealing characteristics of the endobronchial cuffs of commercially used DLTs and bronchial blockers may affect lung-to-lung aspiration. However, these have not yet been compared. In this study, the fluid-sealing characteristics of the endobronchial cuffs of three different commercially available DLTs and two different bronchial blockers were evaluated in the lateral decubitus position using a benchtop model.

## Methods

**Figure 1:**
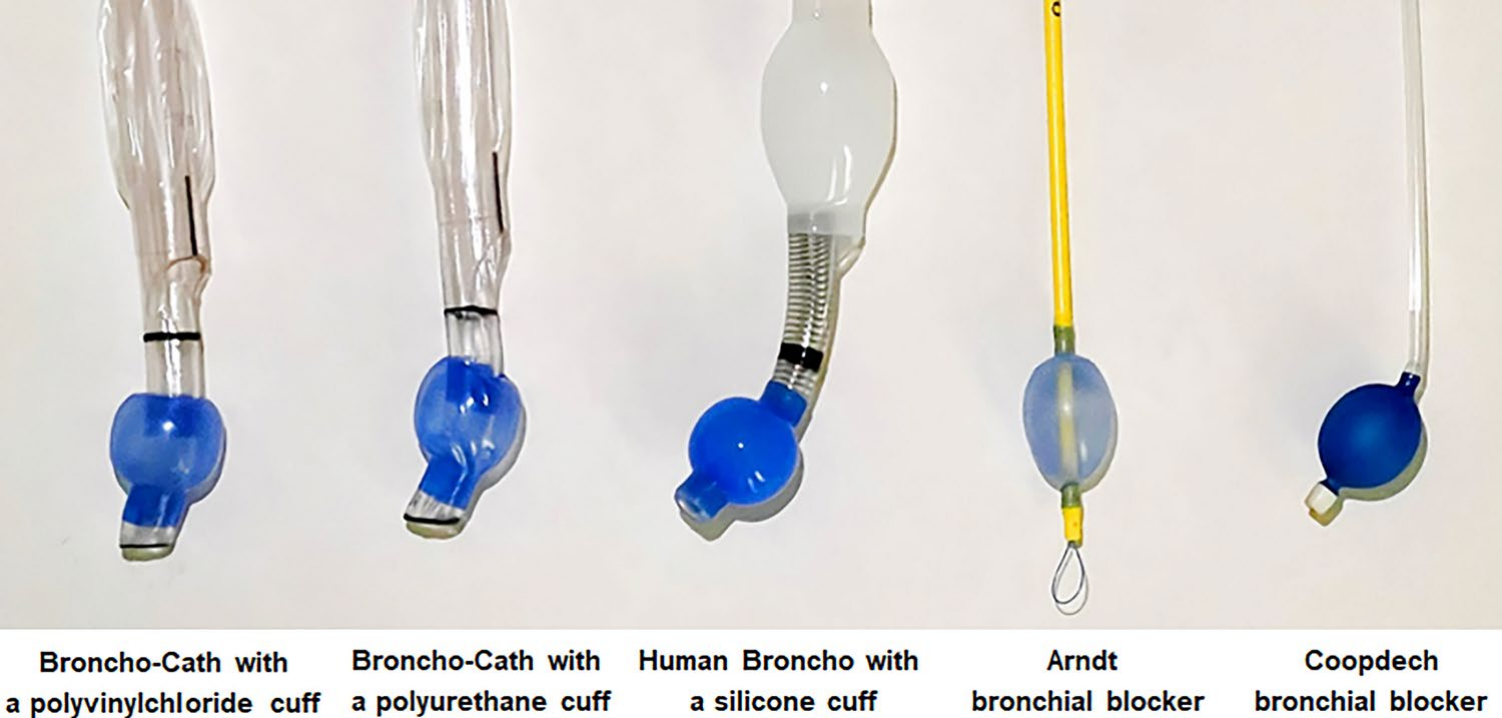
Endobronchial cuffs of different double-lumen endobronchial tubes and bronchial blockers.

This study was an experimental study using a benchtop model, and ethical approval and trial registration were not required. Fluid leakage around the endobronchial cuff was tested using three different left-sided DLTs and two different bronchial blockers: a DLT with an endobronchial cuff made of PVC (Broncho-Cath; Mallinckrodt Medical Inc., Athlone, Ireland), a DLT with an endobronchial cuff made of polyurethane (PU) (Broncho-Cath; Covidien, Athlone, Ireland), a DLT with an endobronchial cuff made of silicone (Human Broncho; Insung Medical Co., Ltd., Seoul, Korea), Arndt bronchial blocker (Cook, Bloomington, IN, USA), and Coopdech bronchial blocker (Daiken Medical Co. Ltd, Osaka, Japan) (Fig. 1).

The artificial tracheobronchial tree was manufactured according to the range of human tracheobronchial tree sizes^8,9^. The inner diameter and length of the trachea were 1.9 and 10 cm, respectively. The inner diameter of the left bronchus was 1.3 cm, and its length was 5 cm. The inner diameter of the right bronchus was 1.6 cm, and its length was 5 cm.

### Study protocol

**Figure 2:**
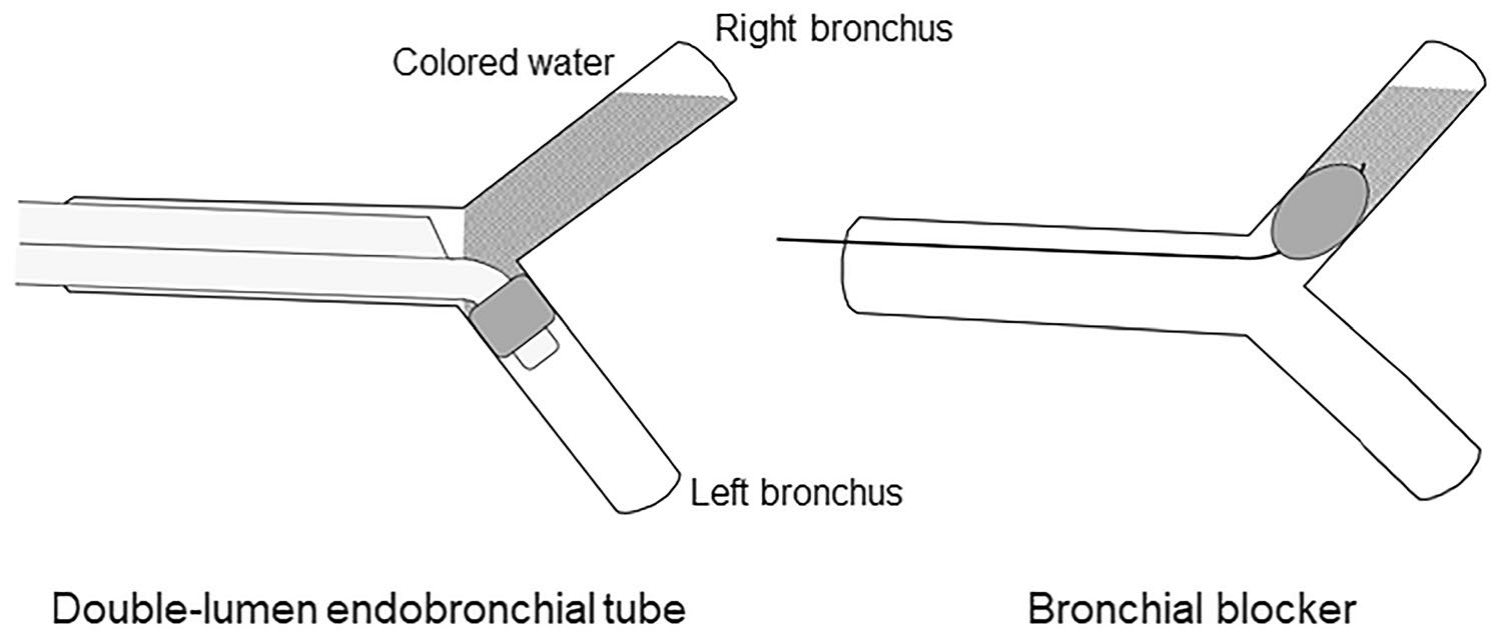
Simulation for lung isolation with the double-lumen endobronchial tube and bronchial blocker in the lateral decubitus position for the surgical management of bleeding from the right lung. The artificial tracheobronchial tree was placed horizontally, with the left bronchus in the dependent position and the right bronchus in the non-dependent position. The bronchus was slightly tilted downwards to prevent water from entering into the tracheal side. The colored water was poured into the right bronchus to simulate the blood flow from the operative side.

The lateral decubitus position for the surgical management of bleeding from the right lung was simulated. The tracheobronchial tree was placed horizontally, with the left bronchus in the dependent position and the right bronchus in the non-dependent position. In the DLT experiments, the tracheobronchial tree was intubated with left-sided DLTs in a simulated lateral decubitus position, and the bronchial tube was inserted into the left bronchus. The endobronchial cuff was inflated to maintain the intracuff pressure at 25 cmH_2_O using a handheld manometer (VBM, Sulz, Germany). The bronchus was slightly tilted downwards to prevent water from entering into the tracheal side. Subsequently, 5 mL of colored water was poured into the right bronchus to simulate the blood flow from the operative side. A water trap was placed to collect the fluid that leaked from the open end of the dependent bronchus. A fluid leakage test around the endobronchial cuff was performed using three different types and sizes (35, 37, and 41 Fr) of DLTs. In the experiments with bronchial blockers, each bronchial blocker (9 Fr) was inserted into the right bronchus, and the endobronchial cuff was inflated with 6–7 mL of air to seal the main bronchial stem. After inflation of the endobronchial cuff within the right bronchus, 5 mL of colored water was poured into the right bronchus to simulate the blood flow from the operative side. A water trap was placed to collect the fluid that leaked from the open end of the dependent bronchus (Fig. 2). According to our pilot study, when 5 mL of water was poured into the non-dependent side, 0.2 mL of fluid was retained within the tracheobronchial tree and endobronchial cuff while the cuff was fully deflated. Therefore, the volume of fluid leakage was monitored and recorded every 30 s until 4.8 mL of poured water was collected. The time to the first leakage around the endobronchial cuff was recorded. The time to 100% leakage around the cuff, defined as the time at which 4.8 mL of the fluid leaked, was also recorded for 24 h. The experiment was repeated six times with new DLTs of each type and size, and new bronchial blockers of each type.

### Statistical analysis

The Statistical Package for the Social Sciences, version 26 (SPSS, IBM Inc., Armonk, NY, USA), was used to conduct the statistical tests. Data are expressed as mean (standard deviation, SD). For each DLT size, the times to first and 100% leakage were compared among the three different types of DLTs using the Kruskal–Wallis test. The time to first leakage between the two different types of bronchial blockers and the time to first leakage between different sizes and types of DLTs and each bronchial blocker were analyzed using the Mann–Whitney U test. The volume of fluid leakage around the endobronchial cuffs over time among the different DLTs and bronchial blockers was analyzed using repeated-measures analysis of variance. *P* < 0.05 were considered statistically significant.

## Results

**Figure 3:**
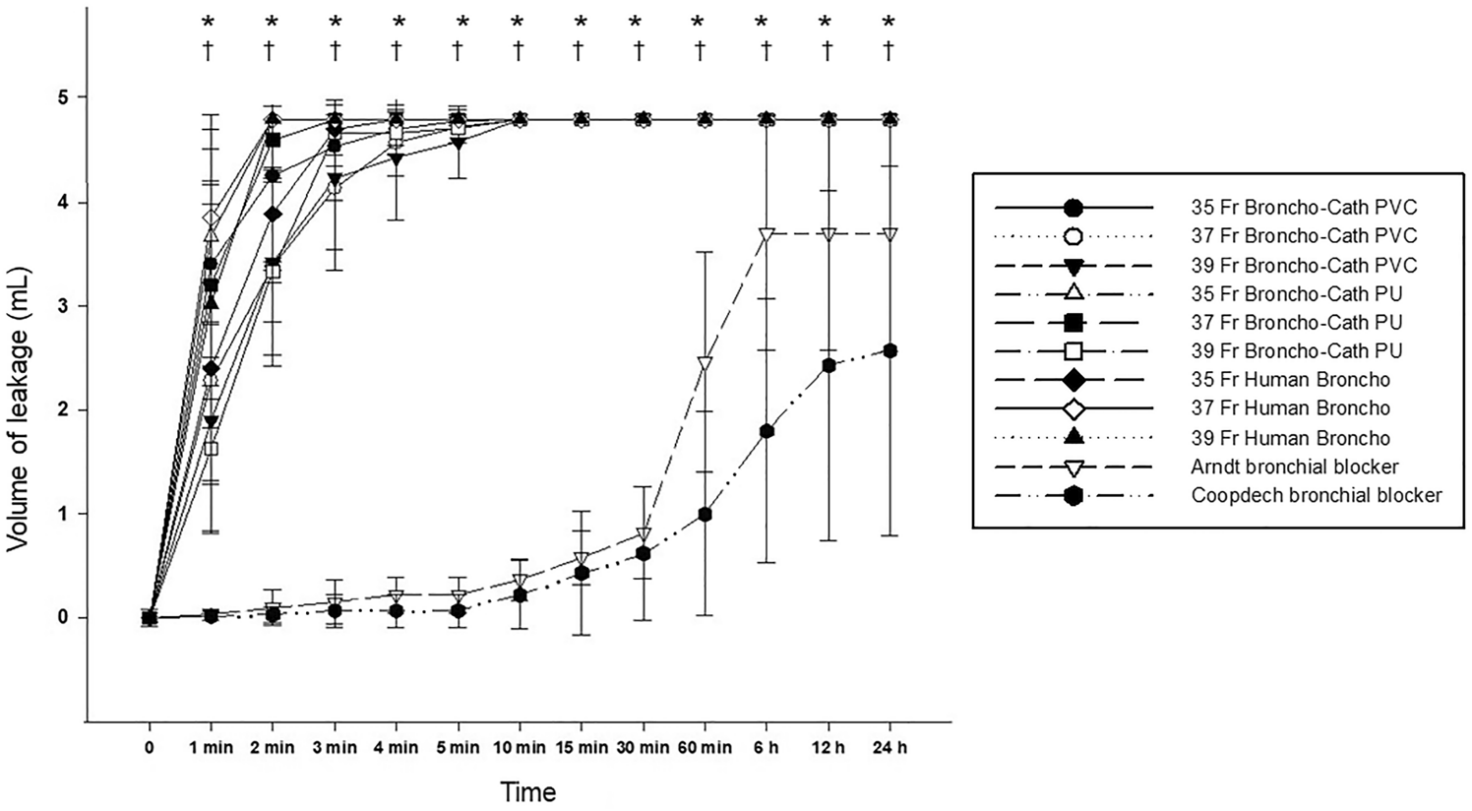
Fluid leakage around the endobronchial cuffs of double-lumen endobronchial tubes and bronchial blockers over time *P < 0.05 versus Arndt bronchial blocker, †*P* < 0.05 versus Coopdech bronchial blocker.

The volume of fluid leakage around the endobronchial cuffs over 24 h is presented in Fig. 3. Each bronchial blocker showed significantly less leakage over time than the other DLTs (*P* < 0.05). Fluid was not fully leaked around the cuffs for 24 h with either bronchial blocker. No significant difference was observed in the amount of leaked fluid over time among the different types and sizes of DLTs. Leaked volume over time was also not significantly different between Arndt and Coopdech bronchial blockers.

The times to first and 100% leakage of water around the endobronchial cuff for different type of DLTs are presented in Table 1. For 35, 37, and 39 Fr DLTs, the times to first and 100% leakage were not significantly different among different type of DLTs (all *P* < 0.05). The time to first leakage did not differ among the three different sizes (35, 37, and 39 Fr) of each type DLT: Broncho-Cath PVC (*P* = 0.389), Broncho-Cath PU (*P* = 0.062), and Human Broncho (*P* = 0.344). The time to 100% leakage was also not different among the three different sizes of each type DLT: Broncho-Cath PVC (*P* = 0.157), Broncho-Cath PU (*P* = 0.052), and Human Broncho (*P* = 0.204).

**Table 1:** Times to first and 100% leakage of water around the endobronchial cuff of double-lumen endobronchial tubes of different types and sizes.

Double-lumen endobronchial tubes	Broncho-Cath (PVC)	Broncho-Cath (PU)	Human Broncho (Silicone)	*P* value
35 Fr				
Time to first leakage (s)	7.5 (7.4)	9.5 (2.3)	8.2 (8.3)	0.466
Time to 100% leakage (s)	143.3 (108.6)	86.7 (19.7)	135.0 (53.6)	0.437
37 Fr				
Time to first leakage (s)	9.2 (4.0)	7.8 (1.5)	4.7 (5.8)	0.246
Time to 100% leakage (s)	236.7 (95.0)	165.0 (62.8)	125.0 (50.9)	0.085
39 Fr				
Time to first leakage (s)	10.5 (6.7)	12.8 (5.5)	6.7 (4.1)	0.111
Time to 100% leakage (s)	250.0 (122.8)	168.3 (40.7)	163.3 (37.2)	0.595

Values are means (SD). *PVC* polyvinylchloride, *PU* polyurethane.

The time to first leakage of water around the endobronchial cuff for different bronchial blockers is presented in Table 2. There was no significant difference in the time to first leakage around the cuffs between the use of Arndt and Coopdech bronchial blockers (*P* = 0.240).

**Table 2:** Times to first and 100% leakage of water around the endobronchial cuff of two different bronchial blockers.

	Arndt	Coopdech	*P* value
Time to first leakage (min)	3.6 (3.2)	14.1 (14.5)	0.240

Values are means (SD).

Each DLT of different types and sizes had a significantly shorter time to the first leakage of water around endobronchial cuffs than Arndt and Coopdech bronchial blockers (all *P* < 0.05) (Table 3).

**Table 3:** Comparisons of time to first leakage of water around the endobronchial cuff among each bronchial blocker and each double-lumen endobronchial tube of different size and type.

Double-lumen endobronchial tubes	*P* value (versus Arndt)	*P* value (versus Coopdech)
Broncho-Cath (PVC) 35 Fr	0.026	0.039
Broncho-Cath (PVC) 37 Fr	0.024	0.039
Broncho-Cath (PVC) 39 Fr	0.023	0.040
Broncho-Cath (PU) 35 Fr	0.026	0.039
Broncho-Cath (PU) 37 Fr	0.024	0.039
Broncho-Cath (PU) 39 Fr	0.023	0.040
Human Broncho 35 Fr	0.026	0.026
Human Broncho 37 Fr	0.024	0.025
Human Broncho 39 Fr	0.023	0.028

*PVC* polyvinylchloride, *PU* polyurethane.

## Discussion

This study showed that bronchial blockers provided a more effective seal against lung-to-lung aspiration than DLTs in the lateral decubitus position for thoracotomy in the benchtop model.

DLTs and bronchial blockers have been used for lung separation during the management of hemoptysis. Bronchial blockers are placed selectively into the bronchus of the bleeding lung through a single-lumen tracheal tube. The correct placement of bronchial blockers requires fiberoptic bronchoscopy guidance. Bronchial blockers are commonly used by physicians for airway protection during the management of hemoptysis^2,10^. In contrast, DLTs are preferred for use in the operating room for thoracotomy because of several advantages, such as suctioning and drainage of blood, the possibility of blind insertion of DLTs, and faster collapse of the surgical side of the lung^4^. Although the correct placement of DLTs requires experience and skill, the placement of DLTs takes less time than that of bronchial blockers, if performed by experienced anesthesiologists.

The endobronchial cuff of bronchial blockers is an inflatable balloon, and a larger volume of air is required to seal the mainstem bronchus than the endobronchial cuff of DLTs. Therefore, it provides tighter seal within the bronchus, thereby preventing lung-to-lung aspiration. In this study, the time to first leakage of water around the endobronchial cuffs was significantly longer with bronchial blockers than that with DLTs. Furthermore, we assessed the volume of leakage of 5 mL of water around the cuff over 24 h, and the water was not fully leaked around the cuffs for 24 h with the bronchial blockers used in this study. However, bronchial blockers are used without the measurement of intracuff pressure, whereas the endobronchial cuffs of DLTs are inflated at the recommended intracuff pressure of 20–30 cmH_2_O^11^. Therefore, the use of bronchial blockers may result in greater mucosal ischemia than DLTs.

The endobronchial cuffs of the DLTs may not provide an effective seal against lung-to-lung aspiration, because fluid or blood would leak through the transverse and longitudinal folds on the bronchial cuff when inflated within the bronchus. We observed that 5 mL of water was fully leaked around the endobronchial cuffs of the DLTs within a few minutes. The intracuff pressure was maintained at 25 cmH_2_O in this study. A previous experimental study showed that higher intracuff pressure (50 and 100 cmH_2_O) provided longer times to first and 100% leakage than the recommended intracuff pressure (25 cmH_2_O). However, even under higher intracuff pressure, the fluid fully leaked around the endobronchial cuffs of DLTs in an hour^12^.

Modification of the cuff material has been suggested to affect the fluid leakage around high-volume and low-pressure cuffs^13^. A previous benchtop study showed that a silicone cuff provided a lower incidence of leakage than conventional high-volume and low-pressure PVC cuffs^14^. Furthermore, an in vitro study suggested that ultrathin polyurethane tube cuffs leak much less and slower than PVC tube cuffs^13^, and a clinical pilot study showed that a polyurethane cuff can reduce the incidence of early postoperative pneumonia^15^. However, a recent systematic review suggested that there is limited evidence to confirm that polyurethane cuffs prevent leakage around the cuff and pneumonia^16^. In this study, lung-to-lung aspiration was investigated using DLTs with endobronchial cuffs made of PVC, polyurethane, and silicone, and the overall results of times to first and 100% leakage of water around the endobronchial cuffs were not significantly different. Gel lubrication of the cuff has also been suggested to prevent aspiration along the longitudinal folds of the cuffs^17^. A previous benchtop study showed that coating a gel lubricant on the endobronchial cuff of DLTs could effectively reduce lung-to-lung aspiration^12^. However, gel lubrication on the endobronchial cuff may be ineffective for prevention of lung-to-lung aspiration in clinical situations, because the coated lubricant on the endobronchial cuff can be removed while passing through the vocal cords and trachea during intubation.

This study has several limitations. First, this is a benchtop study; therefore, the study design might be different from human research. Second, we simulated lung-to-lung aspiration by using a benchtop model, placed in the lateral decubitus position. This would be different from the real clinical situation of hemoptysis. Third, we simulated bleeding from the right lung, and water was poured into the right bronchus to simulate blood. Although we did not simulate bleeding in the left lung, the sealing characteristics of the bronchial cuffs may be similar. Fourth, we used an artificial tracheobronchial tree made of plastic. This differs from the characteristics of the human tracheobronchial tree. Fifth, we used only the left-sided DLTs in the experiments with DLTs, because left-sided DLTs are preferred over the right-sided DLTs because of a greater margin of safety for correct placement during thoracic surgery^18^. Sixth, bleeding was simulated with only 5 mL of colored water in this study. However, hemoptysis or bleeding in real clinical situations may be much more massive.

In conclusion, bronchial blockers prevented lung-to-lung aspiration more effectively than the three different types of DLTs in the lateral decubitus position for thoracic surgery. During surgical intervention for the management of hemoptysis, the use of bronchial blockers can be considered to prevent lung-to-lung aspiration.

## Data Availability

The datasets generated during and/or analyzed during the current study are available from the corresponding author on reasonable request.
